# Unrecognized Transmission Risks of Occult Hepatitis B Virus Infection Among Blood Donors in Central Ethiopia

**DOI:** 10.1155/cjid/3706202

**Published:** 2026-02-05

**Authors:** Gizachew Beykaso, Zeleke Dutamo Agde, Solomon Gebre, Tigist Girma

**Affiliations:** ^1^ School of Public Health, College of Medicine and Health Sciences, Wachemo University, P.O. Box 667, Hossana, Ethiopia, wcu.edu.et; ^2^ Department of Medical Laboratory, College of Medicine and Health Sciences, Wachemo University, P.O. Box 667, Hossana, Ethiopia, wcu.edu.et; ^3^ Department of Virology, Armauer Hansen Research Institute, P.O. Box 1005, Addis Ababa, Ethiopia, ahri.gov.et

**Keywords:** anti-HBc, blood donors, blood safety, central Ethiopia, HBV DNA, OBI

## Abstract

**Background:**

Occult hepatitis B virus infection (OBI) refers to the detection of HBV DNA in blood or liver tissue in individuals who test negative for hepatitis B surface antigen (HBsAg), excluding the early phase of acute HBV infection. This hidden form of infection represents a substantial but frequently underestimated risk to blood transfusion safety, particularly in settings with limited resources. In Ethiopia and other low‐income countries, routine screening of blood donors often depends solely on HBsAg tests, which may overlook symptom‐free carriers with low viral loads. This study was conducted to estimate the prevalence of OBI among HBsAg‐negative blood donors and to assess its potential contribution to undetected HBV transmission in central Ethiopia.

**Methods:**

Five hundred and eighty‐two plasma samples from HBsAg‐negative donors were analyzed. Initial screening for anti‐Hepatitis B core antibodies (anti‐HBc) was carried out using an enzyme‐linked immunosorbent assay (ELISA). Samples that tested positive for anti‐HBc were then analyzed for HBV DNA using the Abbott m2000 automated real‐time polymerase chain reaction (RT‐PCR) system. At the same time, information on sociodemographic characteristics and potential clinical risk factors for HBV exposure was obtained through structured questionnaires. Data were analyzed with SPSS Version 21, and statistical significance was considered at a *p* value of less than 0.05.

**Results:**

In this study, screening of 582 blood donors who were negative for HBsAg revealed that 135 (23.2%) donors, or nearly one‐quarter, showed evidence of previous HBV exposure through anti‐HBc reactivity. Subsequent molecular testing (PCR) identified a small proportion of these individuals carrying HBV DNA, confirming occult infection. Viral loads in all confirmed cases were low. Older donors, particularly those above 35 years of age, demonstrated a markedly higher rate of anti‐HBc positivity. Overall, the occurrence of OBI was estimated at 1.7%, or approximately 17 cases for every 1000 blood donations, highlighting a critical gap in the current HBV screening protocol.

**Conclusion:**

The findings demonstrate that a notable number of blood donors in central Ethiopia carry occult HBV infection, equivalent to 17 cases per 1000 donations. This indicates a concealed yet important risk of HBV transmission through blood transfusion. These results underscore the need to enhance donor screening protocols by incorporating both anti‐HBc testing and nucleic acid testing (NAT) to improve transfusion safety and prevent the spread of undetected HBV.

## 1. Introduction

Hepatitis B virus (HBV) infection remains a significant global public health concern, with an estimated 296 million people chronically infected and more than 820,000 deaths annually, primarily due to chronic liver diseases (CLD), including cirrhosis and hepatocellular carcinoma (HCC) [1–3]. Despite the widespread availability of effective vaccines and antiviral therapies, HBV continues to cause such substantial morbidity and mortality, particularly in low‐ and middle‐income countries [4, 5]. Sub‐Saharan Africa is among the regions with the highest HBV endemicity. Ethiopia is considered hyperendemic, with national HBsAg prevalence estimates ranging from 6% to 10% among the general population, and with studies reporting a pooled prevalence of 7.4% among blood donors [3, 6]. This elevated prevalence heightens the potential for transfusion‐transmitted HBV, especially in these resource‐limited settings where advanced screening technologies are often unavailable and blood donor screening relies exclusively on HBsAg detection [7–9].

Chronic HBV infection is traditionally defined by the persistence of HBsAg in the serum for more than 6 months following acute infection [7, 10, 11]. These chronically infected individuals often present with detectable levels of Hepatitis B envelope antigen (HBeAg) and anti‐HBc, markers indicative of viral replication and past exposure [12, 13]. Anti‐HBc is produced during and following natural infection. And subsequently, it serves as a key serological marker of ongoing or previous HBV exposure. In certain cases, individuals may test positive for anti‐HBc in the absence of both HBsAg and Hepatitis B surface antibody (anti‐HBs), a serologic profile termed isolated anti‐HBc (IA‐HBc) [5, 7, 9, 14]. This profile may represent a false‐positive result, a resolved infection with waning anti‐HBs, or more concerningly, a manifestation of OBI. OBI is defined by the presence of replication‐competent HBV DNA in the liver and/or blood of individuals who are seronegative for HBsAg after acute infection [7]. It represents a clinically silent or cryptic infection that may persist long after apparent clinical resolution [9, 15].

OBI presents a serious threat to transfusion safety [13, 15]. In high‐endemic regions, such as parts of Asia and sub‐Saharan Africa, the prevalence of OBI among HBsAg‐negative blood donors ranges between 10% and 20%, with anti‐HBc positivity reaching up to 50% [16–19]. Blood product from individuals with IA‐HBc or low‐level HBV DNA, especially when a low level of concurrent anti‐HBs antibodies is present, have been shown to transmit HBV to recipients, particularly in immunocompromised individuals, further underscoring the risk posed by OBI [20–24]. In resource‐limited settings like Ethiopia, despite the high HBV endemicity and the recognized risk of transfusion‐transmissible infections, there is a paucity of data on the prevalence and characteristics of OBI among blood donors.

Given the potential for silent HBV transmission through blood transfusion, particularly in settings where screening depends solely on HBsAg detection, it is critical to evaluate the magnitude of OBI among blood donors. Therefore, this study aimed to determine the prevalence of OBI among HBsAg‐negative blood donors in central Ethiopia and to assess its potential implications for transfusion safety and public health policy.

## 2. Materials and Methods

### 2.1. Study Settings

This study was conducted at the Hossana Blood Bank District, which was established in 2014 in Hossana town, the administrative center of central Ethiopia, situated about 232 km southwest of Addis Ababa. The blood bank is the main provider of whole blood and blood components for hospitals and referral facilities in the region, working to fulfill the transfusion requirements of all patients. Each year, it collects more than 9000 units of blood from voluntary, consenting donors. All donated blood is routinely screened for four major transfusion‐transmissible infections: HIV, syphilis, HBV, and HCV using Ag/Ab ELISA for HBsAg and HIV 1/2, and antibody tests for HCV and syphilis. By maintaining these rigorous screening practices, the facility plays a critical role in ensuring transfusion safety and preventing infection‐related complications in the region.

### 2.2. Study Period and Design

A facility‐based cross‐sectional study was conducted from December 2023 to January 2024 to evaluate the risk of OBI transmission among blood donors in central Ethiopia.

### 2.3. Study Population and Eligibility Criteria

The study included voluntary blood donors who tested negative for HBsAg during routine pre‐donation screening. Eligible participants were adults aged 18 years or older, permanent residents of central Ethiopia, who gave informed consent and had no prior history of HBV infection or vaccination within the previous 6 months. Donors with missing demographic information or insufficient blood samples were excluded from the analysis.

### 2.4. Sampling Procedure

The sample size was calculated using a standard sampling formula, resulting in 582 HBsAg‐negative serum samples collected from various sites such as blood banks, schools, and mobile donation sessions between December 2023 and January 2024. Samples were selected through simple random sampling from the daily blood donations that tested negative for HBsAg and were transported to the blood bank laboratory during the study period. The samples were stored at −80°C prior to HBV DNA extraction, amplification, and quantification. Sociodemographic data, including donor sex, age, number of prior donations, donation history, and collection site details, were obtained from blood bank records.

### 2.5. Sample Processing and Detection of OBI

Plasma samples collected were promptly stored at −80°C until further analysis. All blood units that tested negative for HBsAg during routine screening at the district blood bank underwent anti‐HBc testing using the BIORAD Monolisa anti‐HBc ULTRA ELISA kit (France). Samples that tested reactive were retested twice, and a sample was considered positive if at least one of the repeat tests yielded a positive result. For anti‐HBc–positive samples, HBV DNA extraction, amplification, and detection were performed using the ABBOTT m2000sp and m2000rt automated real‐time PCR system, which employs magnetic microparticle reagents to purify nucleic acids from 200 μL of plasma. The assay targets the HBV surface gene sequence and quantifies viral load using an external calibration curve. To ensure reliability, 10% of plasma samples were retested. Viral load quantification was reported in IU/mL, with a conversion factor of 1 IU equaling 3.41 copies of HBV DNA. The Abbott m2000rt system automatically produces and displays quantitative results on its workstation. Samples with detectable HBV DNA but negative for HBsAg were classified as occult HBV infections.

### 2.6. Quality Assurance

Specimen storage and handling were carefully managed to maintain sample integrity. Laboratory procedures were strictly followed by standard operating protocols and manufacturer instructions. Each test run included quality controls provided by the manufacturers, such as high positive, low positive, and negative controls, to verify reagent performance. ELISA results were interpreted based on the cutoff values specified by the test kits.

### 2.7. Data Processing and Analysis

The data was entered and cleaned to quality checks, including verification for completeness, consistency, accuracy, and removal of outliers and duplicates, and extreme values from typing errors, and analyzed using SPSS Version 21. Categorical variables were described using frequencies and percentages, whereas continuous variables were expressed as means with standard deviations. The analysis determined the prevalence of prior HBV exposure (anti‐HBc positive) and occult HBV infection (anti‐HBc positive with detectable HBV DNA). Associations between sociodemographic and clinical factors and seropositivity for anti‐HBc and HBV DNA were evaluated using chi‐square tests and bivariate logistic regression. A stepwise backward elimination method was applied to build the most parsimonious model, removing variables sequentially based on the highest *p* values. After each removal, multicollinearity was assessed using the variance inflation factor (VIF), and Condition Index and Eigenvalues were examined to detect any predictor collinearity. Variables with *p* values less than 0.05 were included in the final model.

## 3. Results

### 3.1. Blood Donors’ Sociodemographic Characteristics

In this study, a total of 582 participants (69.1% male, aged 18–62 years, mean age ± SD = 29.74 ± 8.62 years) were enrolled. Most (69.4%) of the donors were urban residents. In addition, the majority (58.1%) of the donors were married. The majority (80.0%) of donors had a zero to two donation frequency (Table 1).

**Table 1:** Relationship of sociodemographic characteristics with anti‐HBc and HBV DNA positivity in HBsAg‐negative voluntary donors (*N* = 582), Central Ethiopia.

**Variables**	**Donors *N* (%)**	**Anti-HBc+**		**HBV DNA+**	
		***N* (%)**	**p** **value**	***N* (%)**	**p** **value**

*Gender*					
Male	402 (69.1)	81 (20.1)		5 (6.2)	
Female	180 (30.9)	54 (30.0)	0.062	5 (9.3)	0.251

*Age*					
18–29	367 (63.1)	57 (15.3)		3 (5.3)	
30–45	175 (30.1)	59 (33.7)	0.001	5 (8.5)	0.001^∗^
46–65	40 (6.8)	19 (47.5)		2 (10.5)	

*Resident*					
Urban	404 (69.4)	111 (27.5)		9 (8.1)	
Rural	178 (30.6)	24 (13.5)	0.005	1 (4.2)	0.001^∗^

*Educational status*					
Illiterate	67 (11.5)	21 (31.3)		2 (9.5)	
1–8	146 (25.0)	39 (26.7)	0.001	3 (7.7)	0.001^∗^
9–12	162 (27.8)	37 (22.8)		2 (5.4)	
≥ College	207 (35.6)	38 (18.4)		(7.9)	

*Marital status*					
Single	338 (58.1)	58 (16.9)		4 (6.9)	
Married	231 (39.7)	75 (32.1)	0.005	6 (8.0)	0.003^∗^
Divorced	6 (1.0)	1 (16.7)		—	
Widowed	7 (1.2)	2 (28.6).		—	

*Family size*					
≤ 2	142 (24.4)	24 (16.9)		1 (4.2)	
3–5	252 (43.3)	51 (20.2)	0.005	3 (5.9)	0.005^∗^
> 5	188 (32.3)	60 (31.9)		6 (10.0)	

*Blood donation frequency*					
0–2	465 (80.0)	102 (21.2)		8 (7.8)	
3–5	62 (10.7)	15 (24.2)	0.002	2 (13.3)	0.005^∗^
6–8	38 (6.5)	11 (28.9)			
> 8	17 (2,9)	7 (41.2)			

*District*					
Hadiya	116 (19.9)	24 (20.7)		2 (8.3)	
Kambata	74 (17.7)	17 (22.9)	0.005	1 (5.9)	0.001^∗^
Silte	109 (18.7)	28 (25.7)		2 (7.1)	
Halaba	97 (16.7)	22 (22.6)		2 (9.1)	
Yem	76 (13.1)	19 (19.6)		1 (5.3)	
Gurage	110 (18.9)	25 (22.7)		2 (8.0)	

^∗^Significant at *p* value < 0.05.

### 3.2. Prevalence of Occult HBV

In this study, a total of 582 HBsAg seronegative donors’ blood was included, of which 135 (23.2%) were seropositive for HBV anti‐core ELISA test using a BIORAD kit. Among the 135 donors who tested positive for anti‐HBc, HBV DNA was detected in 10 cases. This corresponds to an occult HBV infection prevalence of 7.4% within the anti‐HBc–positive group and 1.72% across all HBsAg‐negative donors (Table 1). The mean HBV DNA load in viremic HBc + donors’ blood was 61  IU/mL, with a range of 44–80  IU/mL.

### 3.3. Associated Factors With Occult HBV

We examined the possible risk factors linked with OBI in the study district. There was a variation in anticore seropositivity between different age groups. It reported that as age increases, the seropositivity for anticore also increases. Among donors aged > 45 years, it was 47.5%, 33.7% among 30–45 years, and 15.3% among donors aged between 18 and 30. Age was identified as one the significant predictor variable, which had a statistically significant association (*p* = 0.001) with anti‐HBc seropositivity. Then again, marital status corresponded with statistical significance (*p* = 0.001) with the occurrence of anticore, where 32.1% of married blood donors had about two times higher anti‐HBc compared to unmarried or single donors whose anti‐HBc was only 16.9%. Similarly, marital status and age were interrelated with anti‐HBc seropositivity (*p* = 0.001), where an increased prevalence (30.0%) was observed among female donors than that in male donors (20.1%), but the difference was not statistically significant. Then again, the increased frequency of blood donation was in line with statistical significance (*p* = 0.001) in the prevalence of anticore, where 41.2% of donors who donated greater than eight times had higher seropositivity of anti‐HBc compared to first‐time donors, whose anti‐HBc was only 21.2% (Table 1).

## 4. Discussion

WHO suggested that blood screening and blood products before transfusion for infectious diseases, such as HBV, HCV, HIV, and syphilis, be obligatory for both developing and developed countries to lessen the risk of transfusion transmission. By 2030, the WHO has also the aims to achieve 100% HBV‐free blood transfusions [3]. However, blood screening for HBV in most African countries, including Ethiopia, exclusively depends on ELISA testing for HBsAg detection, which cannot reveal OBI [9]. Subsequently, there is still a silent risk of HBV transmission through transfusion. Thus, this study revealed the presence of OBI, confirming that HBV infection remains a safety concern in blood donated in this region.

In this study, we investigated 582 HBsAg‐negative serum samples taken from healthy blood donors, and ready for transfusion. We found that 135 anti‐HBc positives or 23.2% out of 582 HBsAg‐negative donors’ blood, which showed a current infection or previous exposure to HBV. To date, there is little evidence from Ethiopia regarding anti‐HBc positivity in blood donors, with the only study conducted in Addis Ababa reporting 14.8% [25]. Compared to this report, the anti‐HBc positivity rate from our study is considerably higher. In our study, this comparative increase in anti‐HBc positivity rate compared to Addis Ababa suggests that our study area is one of the focal points for the prevention and control of HBV in the country.

In non–blood donor population groups, there are studies reported on anti‐HBc seropositivity rates which showed a varied rate of HBV exposure status; 19.5% reported from Hawassa city among children [26], 21.0% from eastern Ethiopia among HIV‐positive adults [27], 26.8% among pregnant women [28], and 6.3% among children from Gondar [29], North Ethiopia. The possible reason for these differences among the studies could be due to the use of different generations of test kits with divergent specificity and sensitivity, in addition to variability in study subjects, occupations, and geographical areas.

In contrast, the reports from African countries demonstrate a wide range of anti‐HBc positivity among blood donors, with elevated rates such as 70.5% in Nigeria [30], 42% in Sudan [31], and 48.7% in Cameroon [32]; lower rates were reported from Egypt 14.2% [33], and one study from Burkina Faso 20.1% comparable rates to our finding [34]. The probable reasons for these differences might be the HBV endemicity in the age variations, population, and screening method.

This study revealed that overall occult hepatitis infection among donors’ blood previously tested HBsAg‐negative and anti‐HBc–positive was found to be 7.4%, which showed blood and blood products comprising anti‐HBc without detectable HBsAg could be unsafe. Evidence from previous studies indicates that anti‐HBc screening alone, without HBV DNA testing, is not recommended in high‐endemic settings due to the risk of unnecessary donor deferral and loss of blood supply [35, 36]. Incorporating this measure could reduce the potential for HBV transmission. This might be reflected as one of the approaches to reduce HBV transmission risk. Likewise, anti‐HBc screening may be a valued procedure to get individuals previously exposed to HBV and potentially bearing substantial risk for HBV reactivation due to immunosuppression [35]. In different nations, using HBsAg and anti‐HBc has been the base for HBV screening, and this has noticeably reduced but did not exclude transfusion‐associated HBV infection.

In Ethiopia, there was no adequate data on OBI prevalence among blood donors except a study in Addis Ababa, which reported 2.8% among blood donors [25], this is due to the absence of molecular tests for blood screening. Whereas the data on OBI among the blood donors is limited, studies carried out among nondonors show that in the Northern region, Gondar, the prevalence was 19.1% among pregnant women [26]; in the Eastern region, Harar, 5.6% among HIV‐negative clients with anti‐HBc seropositive and 6% among HIV patients [27]. In HIV‐positive donors, OBI has been reported to be more common, and hence it is a known risk for its occurrences [37–39] as a result of downregulation of HBsAg synthesis and surface antigen mutation [40]. The variation is also likely due to the endemicity of HBV in the respective local areas and age group differences. Ethiopia introduced universal infant HBV vaccination as part of the pentavalent vaccine in 2007. While this is expected to substantially reduce HBV transmission and future OBI prevalence among donors, its impact on the current donor population remains limited, as most adult donors were born before vaccine implementation. The immunity among transfusion recipients varies depending on vaccination status and prior exposure, and comprehensive national data on postvaccination anti‐HBs coverage in transfusion recipients remain limited.

This study showed that donors’ age, educational status, family size, frequency of donation, and blood groups were found to be increased risks for anti‐HBc and OBI detection, and the argument is also supported by the findings from different studies [41, 42]. The probable reason for older donors having increased anti‐HBc seropositivity than younger donors may be the fact that as age increases, the exposure level to HBV will be higher. It was identified a higher anti‐HBc seropositive and OBI with a decreasing education level. This could be ascribed to the reason that as the education level increases, there is an increased possibility of being observant of preventive actions against HBV infection [19, 20]. Among donors, increased family size was also identified to be associated with higher anti‐HBc seropositive and OBI. This might be because as the family size increases, the probability of household contact increases within the family, which could also increase the cross‐contamination among the family members [41, 43].

### 4.1. Limitations

OBI may be missed in three situations in this study: individuals who are mutually blood seronegative for anti‐HBs and anti‐HBc, seropositive for anti‐HBs but negative for anti‐HBc, and seropositive for anti‐HBc with low‐level HBV DNA which is undetectable. This could alter the actual prevalence of OBI and understate the number of OBI cases. This suggests that OBI may be more widespread than we discovered, which is a cause for concern.

## 5. Conclusions and Recommendations

In this study, we found that around 17 out of 1000 HBsAg‐negative donors had blood deemed “unsafe” after being collected and prepared for transfusion in which the potential transfusion risk from OBI donors is most relevant when anti‐HBs levels are low. Meaning, 7.4% of blood units taken from people who had previously been exposed to HBV were found to have OBI, which can infect blood recipients and keep HBV in circulation through blood transfusions. Given that screening relies solely on HBsAg detection, which is insufficient to eliminate the concealed threat of HBV transfusion transmission, this suggests that the deterrent of OBI has a substantial health impact because of the high possibility of its transmission through blood transfusion. The safety of blood transfusion needs, recipients of blood and blood products, and the elimination of HBV transmission threats in HBV‐endemic areas are all made possible by our results, which support the need for a blood screening strategy that includes anti‐HBc and a complementary sensitive nucleic acid test for detecting HBV DNA by polymerization chain reaction.

## Author Contributions

G.B. made important contributions to the conception and designed the proposal, methodology, data collection, did laboratory work, data analysis, interpretation processes, and wrote the manuscript. Z.D.A., S.G., and T.G. participated in designing the proposal and methodology, data analysis, and interpretation. All authors participated in writing the original manuscript draft and critically reviewed and edited the manuscript.

## Funding

No funding was received for this manuscript.

## Disclosure

All authors read and approved the manuscript version to submit to the current journal and agreed to be accountable for all aspects of the work.

## Ethics Statement

Ethical clearance for the study was secured following a rigorous review and subsequent approval by the Research Ethics Review Committee (RERC) of the College of Medicine and Health Sciences at Wachemo University, under the reference number RERC/012/2023. The research was conducted in full compliance with the ethical principles and procedural standards outlined by the committee, in strict alignment with the National Research Ethics Review Guideline of Ethiopia and in accordance with the Declaration of Helsinki. Moreover, it was ensured that blood banks strictly adhere to a standard protocol requiring written informed consent from donors prior to participation. In accordance with this established practice, the research team obtained the necessary authorization to access and utilize donor information and biological samples, thereby upholding the ethical obligation to respect the autonomy, rights, and confidentiality of all participants involved in the study.

## Conflicts of Interest

The authors declare no conflicts of interest.

## Data Availability

The data analyzed during this study are accessible from the corresponding author on a reasonable request.
